# Optimization of secondary flow path clearance in centrifugal blood pump: a combined numerical and experimental study

**DOI:** 10.3389/fphys.2025.1595588

**Published:** 2025-06-27

**Authors:** Chenying Zhu, Ping Ye, Zhaohua Chang

**Affiliations:** School of Health Science and Engineering, University of Shanghai for Science and Technology, Shanghai, China

**Keywords:** centrifugal blood pump, fluid dynamics, shear stress, hemolysis, secondary flow clearance

## Abstract

**Introduction:**

Blood pumps as mechanical circulatory support (MCS) devices are widely used for patients with ventricular heart failure, and improving blood compatibility remains a key focus for researchers.

**Methods:**

The effects of secondary flow path clearance on the performance and hemocompatibility of a centrifugal blood pump are investigated in this study. A blood pump of the specific design was developed for modeling and computer simulation, and 25 models were generated by varying its internal and outer clearance between 600 and 1,000 μm. This study systematically investigates the hydrodynamic and hemodynamic performance of designed blood pumps through integrated computational fluid dynamics simulations and *in vitro* hydraulic experiments.

**Results:**

Key findings reveal a strong agreement between numerical predictions and experimental data, showing a maximum pressure deviation of 9% for the baseline pump configuration. Furthermore, the effect of the change in clearance on the head and efficiency is less than ±3%. The change in the secondary flow path significantly impacts hemolysis performance.

**Discussion:**

An optimal clearance combination exists in the designed model, and for a specific flow field structure, asymmetric clearance may be an effective means to balance leakage control and turbulence suppression. Although the findings are specific to the pumps examined in this study, they provide valuable insights into the optimal design of blood pumps.

## 1 Introduction

Heart failure (HF) is a significant public health concern, impacting approximately 64 million individuals globally and imposing a considerable strain on healthcare systems due to its elevated mortality and morbidity rates (Shahim et al., 2023). Mechanical circulatory support (MCS) is commonly employed in patients with advanced HF to prolong life expectancy and improve functional capacity, often serving as a pathway to transplantation. Ventricular assist devices (VADs) are mechanical pumps that sustain blood circulation in a failing ventricle for short-term and long-term applications (Khera et al., 2015; Cai et al., 2017). In recent years, centrifugal blood pumps have gained widespread clinical adoption, largely due to the implementation of non-contact magnetic bearings, which minimize mechanical wear and reduce shear-induced blood damage. However, the high-speed rotation of the impeller inherently generates non-physiological shear stress (NPSS), exposing blood to mechanical forces that can damage cells (Thamsen et al., 2015). Excessive shear stress can rupture red blood cells (RBCs) or create membrane perforations once critical stress thresholds are exceeded (Yausda et al., 2000; Li et al., 2013; Shah et al., 2017; Faghih and Sharp, 2019). Research has identified specific shear stress thresholds related to various blood dysfunctions: 9 Pa for the degradation of von Willebrand factor, 50 Pa for platelet activation, and 150 Pa for the destruction of red blood cells. High levels of NPSS (>100 Pa) can cause platelet activation even within a very short exposure time (<1 s) (Chen et al., 2016). Hemolysis is known to follow a power-law relationship with both shear stress magnitude and exposure time. Therefore, precise control of shear stress distribution and blood residence time (RT) is essential in pump design to ensure blood compatibility.

Previous studies primarily focused on the design of the blade and volute of the blood pump, including aspects such as the number of blades, blade angles, and blade thickness, et al. (Li H. et al., 2023; Abbasnezhad and Bakir, 2025; Li Y. et al., 2023; Wu et al., 2021). However, attention has increasingly shifted toward secondary flow path clearances, including axial and radial gaps, which play a critical role in suppressing recirculation, reducing stagnation zones, and improving blood compatibility, especially in magnetically levitated (maglev) pumps where mechanical bearings are absent (Wu et al., 2020). A range of findings have been reported regarding clearance effects. Liu et al. (2019) reported that increasing radial clearance in axial flow pumps led to reductions in head pressure, efficiency, and scalar shear stress (SSS). Puentener et al. (2020) found that a larger radial clearance could significantly improve blood compatibility. Wu et al. (2010) quantitatively calculated pediatric blood pumps, and the results showed that there was an optimal tip clearance, through which hydraulic efficiency could be maximized and hemolysis minimized. While Gil et al. (2022) found that smaller blade tip gaps reduced hydraulic efficiency but increased hemolysis. Goodin et al. (2022) found that reducing secondary clearance had minimal impact on pump head. Rezaienia et al. (2018) demonstrated a unimodal relationship between axial/radial clearances and hemolysis index (HI), highlighting the role of vortices in hemocompatibility. Lv et al. (2024); Lv et al. (2025) conducted numerical simulations to assess the effects of axial blade positions and radial clearance on pump performance and hemolysis. They found that shifting the axial position from the baseline (H = 3.25 mm) to 2.5 and 4.0 mm increased the HI by 12.3% and 24.3%, respectively. Further analysis showed that increasing radial clearance reduced SSS but increased RT, particularly in the bottom clearance. At low flow rates, radial clearance had a greater impact on hemolysis than on thrombosis risk.

A comparative study of CH-VAD and HeartMate III (Wu et al., 2024) showed that while narrow clearances reduce flow disturbances, they can concentrate blood damage near the blades; in contrast, wider clearances enhance secondary flows, increasing flow separation and efficiency loss. Fischer et al. (2025) evaluated 36 clearance configurations and found that even ±10% variations significantly influenced hemocompatibility, concluding that larger, more uniform clearances generally offer better blood compatibility. Despite these advances, existing studies often focus on axial or tip clearances, or only vary one radial clearance parameter at a time. The comprehensive influence of the internal and external radial clearances in the secondary flow path, especially their roles in balancing shear damage and RT, still requires further research.

This study evaluated the design of a centrifugal pump by investigating various parameters, including head, shear stress distribution, and HI. The hydraulic performance was validated through CFD simulations and hydraulic testing. The optimization of the blood pump’s configuration aimed to reduce shear stress and RT, thereby significantly decreasing the incidence of hemolysis. This optimization specifically targeted the radial clearance size of the secondary flow path. The study employed Lagrangian particle tracking to evaluate various parameter models, enabling a comparison of RT and shear stress within the blood pump to predict hemolysis outcomes.

## 2 Materials and methods

### 2.1 Baseline blood pump model

**FIGURE 1:**
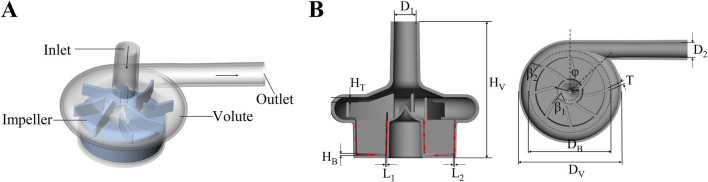
**(A)** Schematic diagram of the centrifugal blood pump structure; **(B)** The axial section and radial section of 3D geometries of the maglev blood pump model.

Figure 1 illustrates a three-dimensional representation of the centrifugal blood pump and its essential structural elements: the inlet, outlet, impeller, and volute. The baseline model was inspired by the general design characteristics of clinically approved centrifugal blood pumps, which are used for short- to medium-term mechanical circulatory support in patients with advanced HF. The inlet and outlet are oriented orthogonally at a 90° angle. The impeller is characterized by a radial, open configuration comprising eight blades, of which four are classified as splitter blades. Prior studies have shown that the incorporation of splitter blades can help regulate the flow field and improve blood compatibility by mitigating flow-induced shear stress and pressure gradients (Wu et al., 2021; Li et al., 2022). A central column within the impeller enhances structural integrity and promotes uniform flow distribution. The impeller is completely supported by an advanced magnetic suspension system, eliminating mechanical contact and reducing wear. This system ensures axial stabilization of the rotor through passive suspension, while radial stability is maintained via a biased magnetic field, facilitating precise control over rotor dynamics. The main design parameters of the blood pump are shown in Table 1.

**TABLE 1:** Main parameters of the blood pump.

Parameter	Parameter description	Value
n	Rotational speed (rpm)	3,000
Q	Flow rate (L/min)	5
H	Design pressure head (mmHg)	300
D_1_	Inlet diameter (mm)	9.5
D_2_	Outlet diameter (mm)	9.2
D_V_	Volute diameter (mm)	64
H_V_	Volute height (mm)	57.5
D_B_	Blade diameter (mm)	49
φ	Volute tongue angle (°)	47.4
N	Number of blades (/)	8
T	Blade thickness (mm)	1.7
β_2_	Blade outlet angle (°)	64
β_1_	Blade inlet angle (°)	56.7
β	Wrap angle (°)	24.5
H_T_	Top clearance (mm)	2
L_1_	Inner clearance (μm)	700
L_2_	Outer clearance (μm)	600
H_B_	Bottom clearance (mm)	1

### 2.2 Optimal experimental design

The selection of the secondary flow path clearance range (600–1,000 μm) was based on two primary considerations. First, geometric constraints inherent to the overall pump head design necessitate compatibility with the magnetic levitation system. In fully maglev pumps, the impeller, housing, and magnetic suspension components must be tightly integrated, which places specific limits on feasible clearance dimensions. Second, the clearance range must support adequate hydraulic performance, including pressure head, efficiency, and flow stability. To identify an optimal configuration with favorable hydraulic and hemolytic performance, an orthogonal experimental design was employed to optimize geometric parameters. Two factors were selected for the secondary clearance design: the inner clearance (L_1_) and the outer clearance (L_2_), each defined at five levels. Using the orthogonal design principle, a total of 25 test combinations were generated, as summarized in Table 2.

**TABLE 2:** Design parameters of clearance size.

Model	Factor	
	L_1_ (μm)	L_2_ (μm)
1	600	600
2	600	700
3	600	800
4	600	900
5	600	1,000
6 (baseline pump)	700	600
7	700	700
8	700	800
9	700	900
10	700	1,000
11	800	600
12	800	700
13	800	800
14	800	900
15	800	1,000
16	900	600
17	900	700
18	900	800
19	900	900
20	900	1,000
21	1,000	600
22	1,000	700
23	1,000	800
24	1,000	900
25	1,000	1,000

### 2.3 CFD analysis

#### 2.3.1 Mesh details and independence analysis

Geometry was initially developed in Solidworks 2023 (Solidworks v2023, Waltham, MA, United States), and imported into Design Modeler (ANSYS, Inc., Canonsburg, PA, United States) for fluid domain extraction. The fluid domain was segmented into three parts: the inlet, the impeller rotation region, and the volute (outlet). Interfaces were created between these sections to ensure seamless data transfer. To enhance the reliability of the simulation, the blood pump’s inlet and outlet were extended. This strategy was employed to minimize the influence of boundary conditions on the simulation results, thereby ensuring that the flow entering the computational domain was fully developed. Unstructured tetrahedral meshes were used for all fluid domains. A more refined meshing strategy was implemented in regions prone to complex flow dynamics or high shear stress, such as near the impeller blades, secondary flow paths, and the volute casing. Specifically, five-layer elements were applied along the walls in these critical areas, enhancing the mesh’s ability to capture boundary layer effects and providing greater accuracy in predicting shear stress distributions. A mesh sensitivity analysis was conducted for the baseline pump, utilizing four grids with sizes of 1.45 million, 2.48 million, 3.95 million, and 5.16 million, respectively.

#### 2.3.2 CFD methods

The software FLUENT 2023R2 (ANSYS, Inc., Canonsburg, PA, United States), was used for numerical simulations. In this model, considering the relatively high shear rates (>100 s^−1^) typically present in rotary blood pumps, the non-Newtonian properties of blood (such as shear thinning behavior, etc.) can be reasonably neglected. Therefore, modeling blood as an incompressible Newtonian fluid is an appropriate simplification, as supported by previous experimental studies (Li et al., 2022; Wu et al., 2024). A constant viscosity of 0.0035 Pa·s and a density of 1,050 kg/m^3^ were adopted in the simulations (Rezaienia et al., 2018). The shear stress transport (SST) k-ω turbulence model was chosen for its high accuracy and robustness in capturing near-wall effects. The impeller’s rotational motion was modeled using the steady “frame motion” approach, and all walls were defined as no-slip boundaries. The semi-implicit pressure-linked equations (SIMPLE) method was utilized to solve the governing fluid equations. A mass flow inlet, corresponding to 0.0875 kg/s (equivalent to a flow rate of 5 L/min), was set for the inlet, while a zero-pressure condition was applied at the outlet. A parametric study was conducted using the workbench to evaluate the hydraulic performance of the centrifugal blood pump, and the inlet and outlet pressure drop and outlet flow were obtained by changing the input parameters (speed and flow). Convergence was deemed achieved when all residuals fell below 10^–3^ and both outlet pressure and flow rate reached a state of stabilization. The governing equations utilized in this analysis are the Navier-Stokes equations, as shown in Equation 1.

$$\rho \left[\frac{\partial u}{\partial t}+\left(u\cdot \nabla \right)u\right]=\mu \nabla^{2}u-\nabla p+\rho g$$ (1)

where u is the velocity field of the fluid, t is the time, μ is the dynamic viscosity, ρ is the density of the fluid, p is the pressure, and g is external body force.

#### 2.3.3 Indicators

Hemolysis is primarily attributed to non-physiological shear stress and prolonged exposure. An increase in shear stress can result in damage to red blood cells. The shear stress tensor is calculated based on the velocity field obtained from a three-dimensional numerical simulation. Equations 2, 3 are the corrected formula for calculating the SSS (Faghih and Keith Sharp, 2016):

$$\tau_{scalar}={\left[\frac{1}{12}\sum {\left(\tau_{ii}-\tau_{jj}\right)}^{2}+\frac{1}{2}\sum {\tau_{ij}}^{2}\right]}^{\frac{1}{2}}$$ (2)

$$\tau_{ij}=\mu \left(\frac{\partial U_{i}}{\partial x_{j}}+\frac{\partial U_{j}}{\partial x_{i}}\right)$$ (3)

where $U_{i}$ is blood velocity and μ is blood viscosity.

The Lagrange method was applied to track particle trajectories between the inlet and outlet of the device. The HI values were integrated along each particle trajectory and averaged to determine the total HI value of the blood flowing through the device (Garon and Farinas, 2004). Equation 4 is the specific calculation formula. The HI along all pathlines were considered representative of the overall HI for the pump.

$$HI=\frac{1}{N_{P}}\sum_{P=1}^{N_{P}}C{\left(\sum_{inlet}^{outlet}∆t\tau^{\alpha /\beta }\right)}^{\beta }$$ (4)

where $C=1.228\times 10^{-5};\alpha =1.9918;\beta =0.6606$ for ovine blood (Ding et al., 2015). τ is the SSS, ∆t is the RT. $N_{P}$ is the number of track particles.

### 2.4 Validation

**FIGURE 2:**
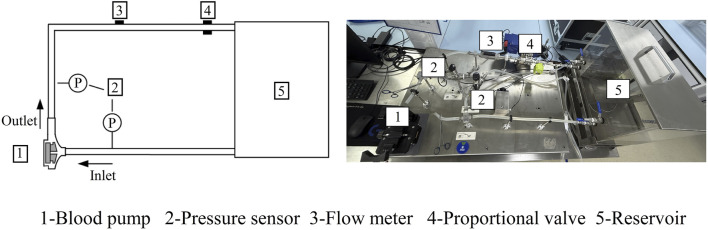
Schematic and photograph of the flow loop used to simulate hydraulic properties.

Experimental tests were carried out to evaluate the hydraulic performance of the baseline pump. The experimental setup is depicted in Figure 2. The pump head was fabricated from transparent polycarbonate (PC) material, with both internal and external surfaces polished to minimize flow resistance. A water–glycerol mixture (viscosity of 0.0035 Pa·s at 24°C) was used to match blood viscosity. Although this Newtonian fluid does not capture the shear-thinning behavior of real blood, prior studies suggest that in high-shear regions (>100 s^−1^), the approximation remains valid for hydraulic performance validation. The inlet and outlet pressures were measured using digital pressure sensors (model GP-M001, Keyence), while an ultrasonic flow meter (model FD-XS20, Keyence) was used to capture the volumetric flow rates. To assess the pump’s performance under different operating conditions, various experimental scenarios were established by adjusting the pump’s rotational speed and the proportional valve on the outflow tubing. These adjustments allowed for a comprehensive evaluation of the pump’s hydraulic characteristics. It was assumed that maintaining the rotational stability of the motor shaft would not significantly affect the fluid dynamics. The pump was tested across a range of rotational speeds, from 2,500 to 3,500 rpm, with 24 operational tests conducted to record pressure head and flow rate data.

**FIGURE 3:**
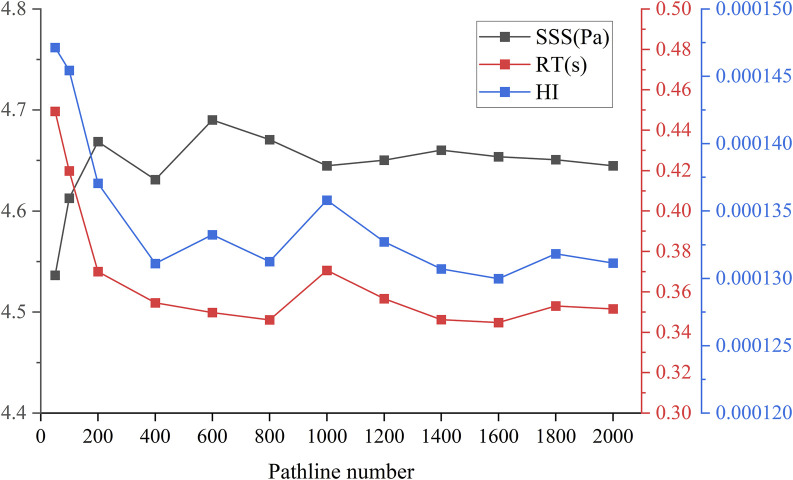
Pathline independence: Sensitivity analysis of particle size number with SSS(Pa), RT(s) and HI.

As illustrated in Figure 3, a range of particle trace numbers was selected to assess their predictive accuracy for hemolysis. It was observed that when the particle trace number reached 1,600, the hemolysis value remained relatively stable, even with further increases in the number of traces. In order to minimize the calculation error as much as possible, the subsequent HI prediction experiments utilized 2,000 particle traces for calculations.

## 3 Results

### 3.1 Mesh independence analysis

A mesh independence analysis was conducted to validate the adequacy of mesh quality for precise computational results (see Table 3). Four different mesh resolutions, ranging from coarse to fine, were created, and corresponding fluid dynamic simulations were performed to assess the impact of mesh density on the pump head. The predicted pressure head stabilized beyond 2.5 million elements, with Case 3 (3.95 million elements) yielding an error of less than 0.4% compared to the finest mesh. Considering both computational efficiency and accuracy, the third mesh configuration was selected for all subsequent analyses. The grid elements for different pump models were kept at around four million.

**TABLE 3:** Results of Mesh independence analysis.

Case	Mesh number ( $\times 10^{6}$ )	Average Y+ of all faces	Head (mmHg)	Error of head (%)
1	1.45	9.07	320.75	4.56
2	2.48	0.361	318.09	3.70
3	3.95	0.353	305.61	0.37
4	5.16	0.327	306.75	-

Error of head (%), defined as |head* − head_4_|/head_4_ * 100, where head_4_ is the head predicted for case 4; head* represents the head of each case.

### 3.2 Experiment validation of hydraulic performance

**FIGURE 4:**
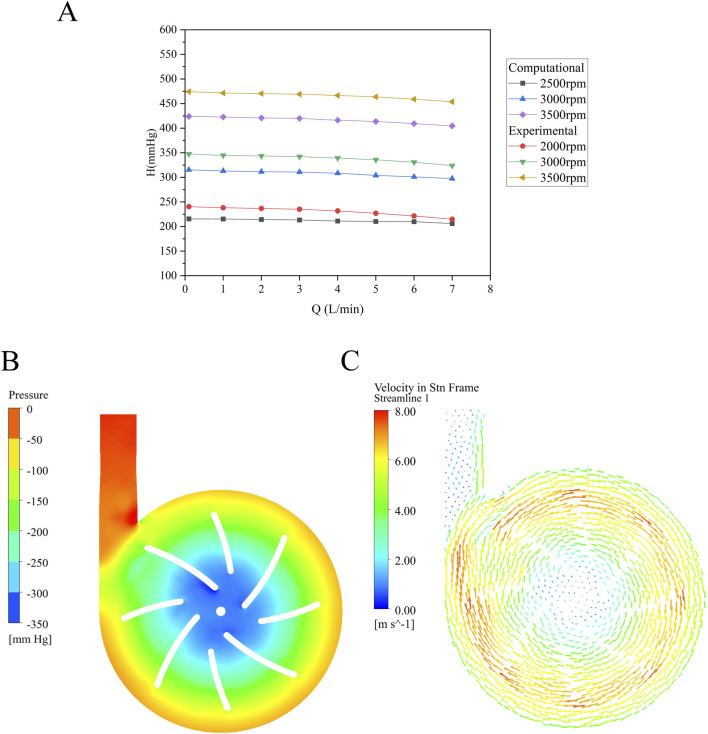
**(A)** H-Q curve (the simulated head and measured head at different rotational speeds) of the baseline blood pump; **(B)** Pressure distribution within the baseline pump at 3,000 rpm and 5 L/min; **(C)** Velocity vector field showing flow direction and magnitude.

The relationship between the inlet-outlet pressure difference and flow rate (H-Q curves) at a given rotational speed is a key parameter for assessing the hydraulic performance of a blood pump. The simulated results at various rotational speeds were validated against experimental data as shown in Figure 4A. The overall trend of the H-Q curve exhibits a relatively smooth profile, consistent with the characteristics of a centrifugal pump. The maximum error of approximately 9% observed at 3,500 rpm and 0.1 L/min represents the difference between CFD simulation results and experimental measurements.

To further validate the flow characteristics within the pump, the pressure and velocity vector fields of the baseline model were extracted at a flow rate of 5 L/min and a rotation speed of 3,000 rpm. These measurements were taken from the cross-section, as illustrated in Figures 4B, C. Localized low-pressure regions are observed near the leading edge of the blades and the center column. Pressure increases radially from the center toward the periphery, corresponding to the acceleration of fluid outward, which results in a pressure rise. The volute exhibits a uniform distribution, with a pronounced pressure increase observed near the outlet.

### 3.3 Influence of clearance on hydraulic performance

The influence of secondary flow path clearances on hydraulic performance was systematically assessed across 25 orthogonal design combinations (Table 4). The changes in clearance affected the pump volume minimally (<3%), and the variations in pressure head and efficiency remained within ±3% and ±5%, respectively, suggesting a limited influence of secondary flow path clearance adjustments on pump hydraulic performance. Compared with the baseline pump, several configurations significantly improved the hydraulic head and efficiency. Model 16 achieved the highest efficiency (+5.16%), while Model 21 attained the maximum pressure head (+3.10%). However, certain designs, such as Model 13, led to performance deterioration (efficiency −4.53%), indicating sensitivity to structural changes. An increase in clearance results in a higher leakage flow within the secondary flow path.

**TABLE 4:** Comparison of hydraulic properties of different clearance sizes.

Model	Volume (V) (mL)	V_error_ (%)	Head (H) (mmHg)	H_error_ (%)	Efficiency (η) (%)	η_error_ (%)	Leakage flow (kg/s)	Leakage portion (%)
1	32.91	−0.21	310.87	1.72	42.75	1.83	0.00143	1.63
2	33.10	0.37	307.23	0.53	41.50	−1.14	0.00059	0.67
3	33.30	0.96	303.33	−0.75	41.11	−2.07	0.00060	0.68
4	33.50	1.60	305.31	−0.10	41.52	−1.10	0.00116	1.32
5	33.71	2.22	307.96	0.77	41.70	−0.67	0.00048	0.55
6 (baseline pump)	32.98	—	305.61	—	41.98	—	0.00052	0.59
7	33.17	0.59	305.52	−0.03	41.67	−0.74	0.00081	0.93
8	33.38	1.21	301.63	−1.30	41.02	−2.29	0.00065	0.74
9	33.58	1.82	310.93	1.74	42.20	0.52	0.00119	1.36
10	33.78	2.44	313.30	2.52	43.47	3.54	0.00049	0.56
11	33.05	0.21	301.04	−1.50	40.98	−2.38	0.00059	0.68
12	33.24	0.80	301.42	−1.37	40.52	−3.48	0.00047	0.53
13	33.45	1.42	301.87	−1.22	40.08	−4.53	0.00084	0.96
14	33.65	2.03	305.06	−0.18	41.21	−1.82	0.00091	1.04
15	33.85	2.65	305.89	0.09	41.96	−0.05	0.00047	0.53
16	33.19	0.64	304.23	−0.45	44.15	5.16	0.00113	1.29
17	33.31	1.02	301.88	−1.22	40.81	−2.79	0.00054	0.62
18	33.52	1.63	310.10	1.47	40.81	−2.79	0.00108	1.24
19	33.72	2.25	307.63	0.66	42.93	2.27	0.00090	1.02
20	33.92	2.87	304.03	−0.52	40.81	−2.78	0.00085	0.98
21	33.19	0.64	315.10	3.10	42.16	0.42	0.00055	0.62
22	33.38	1.23	301.15	−1.46	40.80	−2.81	0.00066	0.75
23	33.59	1.84	307.34	0.57	43.18	2.85	0.00070	0.80
24	33.79	2.46	299.81	−1.90	41.05	−2.22	0.00072	0.83
25	33.99	3.08	304.13	−0.48	40.26	−4.10	0.00115	1.31

V_error_, defined as (V* − V_baseline_)∕V_baseline_*100; V* represents the volume of each model; H_error_, defined as (H* − H_baseline_)∕H_baseline_*100; H* represents the pressure head of each model; **η**_error_, defined as (η* − **η**_baseline_)∕**η**_baseline_*100; η* represents the efficiency of each model. Ratio of Leakage flow (%), defined as Q_leakage flow_/Q_inlet_. The leakage flow is defined as the flow rate at the cross-section of the inlet of the secondary flow path.

### 3.4 Influence of clearance on SSS

**FIGURE 5:**
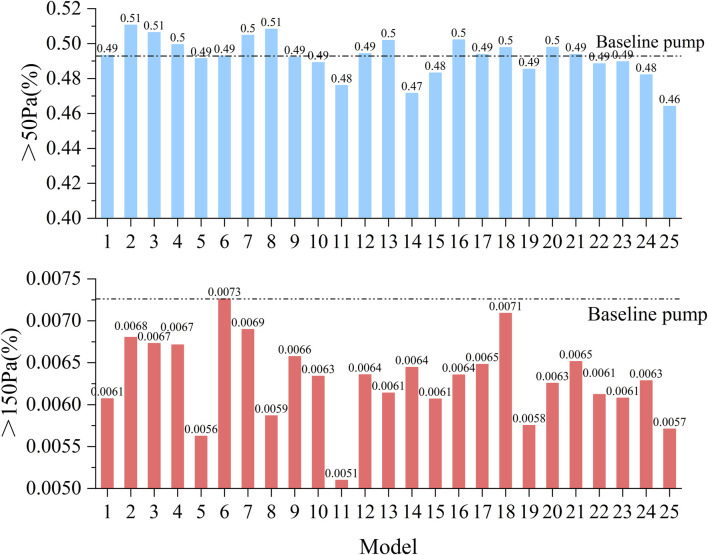
Statistical comparison of the volume proportions exceeding shear stress thresholds of 50 and 150 Pa across all 25 pump configurations.

High SSS areas increase the risk of thrombosis, and identifying high SSS areas in the blood pump is important for predicting the probability of thrombosis. It was classified into three levels: (1) above 9 Pa: induced von Willebrand factor cleavage; (2) above 50 Pa can induce platelet activation; (3) above 150 Pa, associated with hemolysis risk (Fraser et al., 2012). Figure 5 illustrates the volume ratios of SSS exceeding 50 and 150 Pa across the 25 models. The volume fraction corresponding to high shear stress (>50 Pa) ranges from approximately 0.46%–0.51%, indicating that although high shear regions occupy a relatively small portion of the total volume, they still pose a potential risk of hemolysis. For regions with extremely high shear stress (>150 Pa), the volume fraction is even smaller, consistently below 0.0073%. Among all models, Model 2 exhibits the highest proportion of regions with SSS greater than 50 Pa, reaching 0.51%, whereas Model 25 shows the lowest proportion at 0.46%. Regarding the extremely high shear stress (>150 Pa), the baseline pump displays the highest volume ratio of 0.0073%, while Model 25 has the lowest at 0.0057%.

**FIGURE 6:**
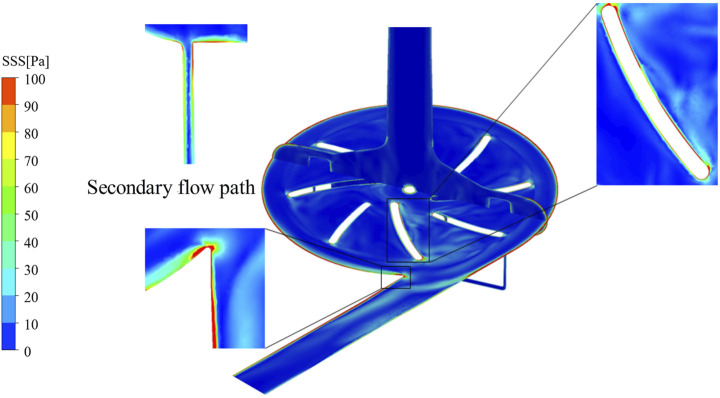
Three-dimensional distribution of SSS(Pa) for the baseline pump.

To further illustrate the spatial characteristics of shear stress distribution, the baseline pump model (Model 6) was selected for detailed visualization of SSS, as shown in Figure 6. This configuration exhibits extensive regions exceeding the hemolysis threshold of 150 Pa, with localized high-shear zones observed near the leading edges of the impeller blades, the volute tongue, and the secondary flow path area.

### 3.5 Orthogonal analysis and optimal design

**FIGURE 7:**
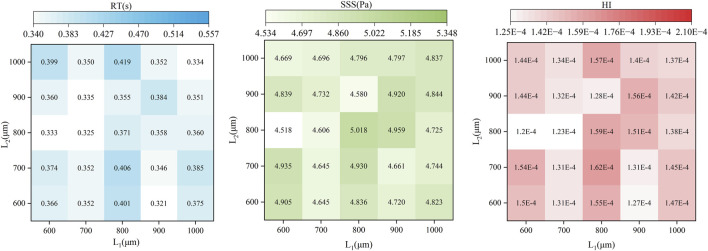
Heatmaps showing the distribution of RT(s), SSS(Pa), and HI across 25 pump configurations with varying secondary clearance combinations.

**FIGURE 8:**
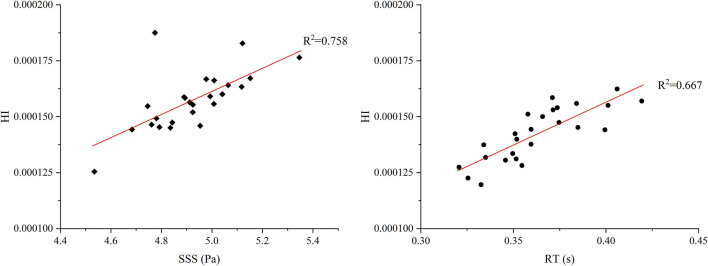
Linear regression analysis of HI with two key hemodynamic parameters: RT(s) and SSS(Pa).

In Figure 7, the impact of the radial clearance size in the secondary path on the average SSS, average RT, and HI value of particle paths is compared. The RT heat map indicates that the overall RT values range from 0.32 to 0.42 s. With the increase of L_2_, especially when L_1_ is small (such as 600 μm), RT tends to increase initially and then decrease. In contrast, when L_1_ and L_2_ is larger (such as 900–1,000 μm), the distribution of RT becomes more uniform. The maximum RT occurred at L_1_ = 800 μm and L_2_ = 1,000 μm (0.419 s), while the minimum value occurred at L_1_ = 900 μm and L_2_ = 600 μm (0.321 s). The SSS heat map shows relatively small variation across the parameter space, with most values falling between 4.5 and 5.0 Pa. The SSS value reaches the maximum (5.018 Pa) at L_1_ = 800 μm and L_2_ = 800 μm, whereas the minimum (4.518 Pa) at L_1_ = 600 μm and L_2_ = 800 μm. When L_1_ or L_2_ is fixed, SSS does not exhibit a clear monotonic trend but instead shows nonlinear fluctuations, indicating that L_1_ and L_2_ have a complex coupling effect on shear stress. When L_1_ = 600 μm and L_2_ = 800 μm, the HI is the lowest at 0.00012%. While the highest HI (0.00016%) appears at L_1_ = 800 μm and L_2_ = 700 μm. Compared with the baseline pump, the HI shows a minimum reduction of 8.76% and a maximum increase of 23.86%. Overall, HI tends to increase slightly with higher SSS; however, the correlation is not strictly consistent. To quantitatively assess the relative contributions of RT and SSS to hemolysis, a linear regression analysis was performed between the predicted HI and each of the two parameters across 25 simulation cases (Figure 8). The results show that HI exhibited a stronger correlation with SSS (*R* ^2^ = 0.758) than with RT (*R* ^2^ = 0.667), indicating that shear-induced damage is more predictive of hemolysis than particle RT.

**FIGURE 9:**
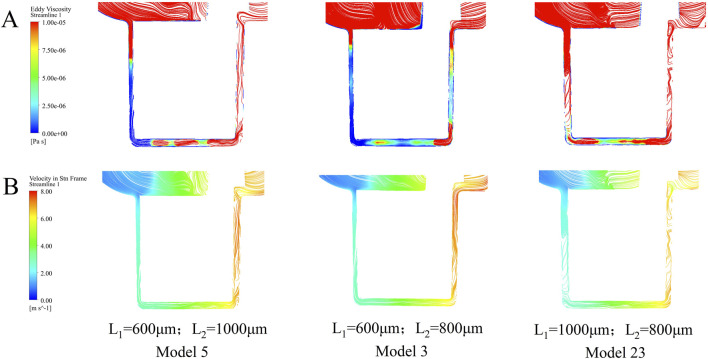
The streamlines of the three selected models are colored using **(A)** turbulence intensity and **(B)** velocity.

Turbulent viscosity serves as a key indicator for characterizing the intensity of turbulence within the pump. Figure 9 present the streamlines of three representative pump models, with color maps indicating turbulent viscosity and velocity, respectively. Model 23 exhibits substantially higher turbulent viscosity throughout the pump compared to Models 3 and 5, with Model 5 showing moderately higher values than Model 3. These results indicate that compared with the optimal model, increasing either the inner (L_1_) or outer (L_2_) secondary clearance leads to a notable increase in turbulence intensity, particularly within the secondary flow path regions.

Calculate the RT, SSS, and HI of the blood pump by 25 groups of orthogonal designs. Table 5 presents the mean values of the three indicators calculated using Equation 5 at each level of each factor.

$$k_{i}=\frac{1}{N_{i}}K_{i}=\frac{1}{N_{i}}\sum_{j=1}^{N_{i}}y_{i},j$$ (5)

**TABLE 5:** The sum and mean of each level of every factor.

Factor	Level	Results		
		Average RT(s)	Average SSS(Pa)	Average HI
L_1_	600	0.366	4.773	0.000142
	700	0.347	4.721	0.000134
	800	0.390	4.832	0.000152
	900	0.352	4.811	0.000141
	1,000	0.361	4.795	0.000142
R		0.043	0.111	0.000018
Optimal level		700	700	700
L_2_	600	0.363	4.786	0.000142
	700	0.367	4.775	0.000143
	800	0.351	4.790	0.000140
	900	0.360	4.775	0.000141
	1,000	0.375	4.806	0.000146
R		0.024	0.031	0.000007
Optimal level		800	700/900	800

The range R in Equation 6 is the difference between the maximum and minimum values of a single prediction result.

$$R=\max\left(k_{1},k_{2},{\ldots ,k}_{i}\right)-\min\left(k_{1},k,{\ldots ,k}_{i}\right)$$ (6)

The R value reflects the impact of each factor on the optimization; a larger R value indicates a greater influence. As shown in Table 5, the influence of L_1_ on average SSS, average RT, and average HI is consistently greater than that of L_2_. The minimum average SSS values for factors L_1_ and L_2_ occur at 700 and 800 μm, respectively. For average RT, the minimum values correspond to 700 μm for L_1_ and 700 μm or 900 μm for L_2_. When HI is taken as the final optimization objective, the optimal design parameter combination is L_1_ = 700 μm and L_2_ = 800 μm.

## 4 Discussion

This study presents a pump head suitable for a fully maglev non-contact centrifugal pump designed for use in VADs. The pump head’s hydraulic and hemodynamic performance was assessed through a combination of CFD simulations and experimental validation. The use of an unstructured tetrahedral mesh model in conjunction with the k-ω shear stress transport turbulence model provided a detailed understanding of the flow dynamics within the pump. The grid independence analysis method was used to evaluate the variation in pump head pressure with different grid numbers. Finally, about four million grid cells were selected for CFD calculation and analysis. After conducting the hydraulic test, the pressure-flow curve of the centrifugal pump was created. The results indicated that the simulated head pressure was consistent with the measured head pressure. The pressure of the centrifugal pump changed gently with the flow rate during the whole working process, and there was no pressure distortion, indicating that the flow field design of the pump was reasonable.

Our results demonstrate that variations in secondary clearance (L_1_ and L_2_) have a limited influence on overall hydraulic performance, with pressure head and efficiency fluctuating within ±5%. This aligns with the findings by Fischer et al. (2025), who reported that axial and radial clearance size variations (0.5–3 mm) had a relatively small effect (±10%) on key performance indicators. However, these clearance modifications significantly affect blood compatibility indicators, particularly HI, shear stress distribution, RT, and turbulence intensity. Among all tested configurations, the combination of L_1_ = 600 µm and L_2_ = 800 µm yielded the lowest HI, demonstrating an 8.76% improvement over the baseline.

Analysis of shear stress and flow patterns revealed that hemolysis is primarily driven by localized high shear stress zones and prolonged blood residence in recirculatory regions. The average RT of the particles varies with the increase of the clearance in the secondary flow path. A common trend observed is that if L_1_ or L_2_ is fixed and another clearance size is increased, the average RT first shows a decrease, and then continues to increase as the clearance further increases. Compared to the model with the lowest HI (L_1_ = 600 μm, L_2_ = 800 μm), increasing the outer clearance L_2_ to 1,000 μm while keeping L_1_ fixed led to a rise in RT from 0.333 to 0.399 s, whereas the SSS showed only a slight change (from 4.518 to 4.669 Pa). As shown in Figure 9, this increase in RT is primarily attributed to elevated turbulence intensity within the secondary flow path. In particular, intensified turbulence in the bottom gap region prolongs blood retention, ultimately contributing to a significant increase in the HI. Regression analysis confirmed a stronger correlation between HI and SSS (*R* ^2^ = 0.758) than RT (*R* ^2^ = 0.667), reinforcing the dominance of shear-induced hemolysis mechanisms.

Our findings align with previous studies by Rezaienia et al., 2018) and Fischer et al. (2024), who observed that axial and radial clearances affect hemolysis through their impact on secondary flows and vortices. However, our study extends these insights by employing a denser parametric matrix (25 combinations) and integrating Lagrangian particle tracking to predict HI more accurately. By designing radial clearances of different sizes and predicting their impact on hemolysis, it was found that there is an optimal range for the radial clearance size. Hemolysis is strongly influenced by high shear stress and flow recirculation, and proper clearance selection can mitigate these effects without significantly compromising hydraulic performance.

Studies have shown that hemolysis is closely related to high shear stress and flow recirculation within the pump, and the design of the secondary flow path clearance can significantly influence these factors. Although experiments have partially verified the simulation and numerical predictions presented in this study, there are still some parts that need to be continued. While this work examined the effects of selected discrete combinations of inner (L_1_) and outer (L_2_) clearance sizes on hemodynamic and hemolytic performance, a comprehensive sensitivity analysis across a continuous range of gap sizes was not feasible due to computational constraints. Future studies will aim to conduct higher-resolution parametric analyses and incorporate uncertainty quantification to more thoroughly assess the influence of clearance variations on pump performance and blood compatibility. Moreover, the current numerical model employed a steady-state CFD approach. However, transient simulations are necessary to capture time-dependent shear stress fluctuations and hemolysis dynamics, which are important for evaluating the pump’s washing efficiency and long-term blood compatibility (Fang et al., 2023). Incorporating unsteady flow analysis in future work will enable a more accurate and holistic understanding of pump performance under physiological conditions. This study also adopted a Newtonian fluid model to simplify blood rheology. Nonetheless, it is well recognized that blood exhibits non-Newtonian shear-thinning behavior, particularly in low shear regions where viscosity can vary significantly. Future simulations will adopt a Carreau–Yasuda model to more accurately represent the shear-dependent viscosity and improve the predictive accuracy of hemolysis risk. Finally, further *in vitro* validation, especially direct quantification of hemolysis, is essential to confirm the numerical findings and assess the clinical viability of the pump design. In particular, prolonged RT in low-shear regions not only exacerbates hemolysis but also elevates thrombogenic risk due to platelet activation and aggregation. Therefore, future research should also include thrombogenicity evaluation to provide a comprehensive assessment of the pump’s blood compatibility.

## Data Availability

The raw data supporting the conclusion of this article will be made available by the authors, without undue reservation.
